# Annexin A2 negatively regulates IFN-β production through targeting the RLR signaling pathway

**DOI:** 10.1128/jvi.01945-25

**Published:** 2026-01-16

**Authors:** Hongyang Liu, Mengdi Xue, Chunying Feng, Jimin Yu, Guangqiang Ye, Kunli Zhang, Li Huang, Changjiang Weng

**Affiliations:** 1 Division of Fundamental Immunology, State Key Laboratory of Animal Disease Control and Prevention, Harbin Veterinary Research Institute of Chinese Academy of Agricultural Sciences 111613 , Harbin, China; 2 Heilongjiang Provincial Key Laboratory of Veterinary Immunology 559566, Harbin, Heilongjiang, China; Loyola University Chicago - Health Sciences Campus, Maywood, Illinois, USA

**Keywords:** Annexin A2, type I IFN, MDA5

## Abstract

Annexin A2 (ANXA2) is known to promote the replication of diverse RNA viruses, often through interactions with specific viral proteins. However, whether it also employs a broad-spectrum, virus-independent strategy to facilitate viral replication remains unclear. Here, we identify ANXA2 as a novel negative regulator of the host antiviral response by targeting the RIG-I-like receptor (RLR) signaling pathway. We demonstrate that overexpression of ANXA2 suppresses type I interferon (IFN) production induced by RNA viruses or poly(I:C), whereas ANXA2 deficiency enhances IFN production and restricts viral replication both *in vitro* and *in vivo*. Mechanistically, ANXA2 functions as a scaffold protein that concurrently disrupts two critical steps in RLR signaling: it impedes the recruitment of MAVS by MDA5 through interacting with the CARD domain of MDA5, and it inhibits the MAVS-TRAF3 interaction by binding to the linker region of MAVS. Consequently, ANXA2 deficiency leads to enhanced type I IFN production in mice, which effectively restrains replication of encephalomyocarditis virus and vesicular stomatitis virus. Collectively, our study uncovers a novel and broad-spectrum immunomodulatory function of ANXA2, wherein it dampens antiviral innate immunity by sabotaging key protein-protein interactions (MDA5-MAVS or MAVS-TRAF3) within the RLR pathway, thereby presenting a potential target for developing antiviral strategies.

**IMPORTANCE:**

Subsequent to RNA viral infection, a series of complex cascade reactions are initiated, leading to the production of type I interferons and, consequently, the resistance of the organism to viral infection. This study elucidates the function of Annexin A2 (ANXA2) as a novel key negative regulator in the host antiviral immune response. Mechanistically, ANXA2 achieves its inhibitory effect by disrupting critical signaling steps in the RLR pathway, specifically interfering with key interactions between MDA5 and MAVS, as well as between MAVS and TRAF3. These findings are significant in that they reveal an unknown mechanism by which viruses exploit host proteins to evade immunity, and they position ANXA2 as a potential therapeutic target for developing novel antiviral strategies. The validation of these findings in an ANXA2-deficient mouse model, which exhibits enhanced interferon production and restricted viral replication, serves to further reinforce the physiological relevance of these observations.

## INTRODUCTION

Annexin A2 (ANXA2), also known as calpactin I or lipocortin II, is a multifunctional protein that can reversibly bind to phospholipid membranes and calcium ions (Ca^2+^) (1). ANXA2 belongs to the membrane scaffold and binding protein family and is primarily expressed in the plasma membrane and intracellular vesicles (2, 3). It has been reported that ANXA2 participates in the replication process of various viruses. For example, ANXA2 interacts with the matrix (M) protein of viruses, such as measles virus and bovine ephemeral fever virus, which is situated on the inner surface of the virus envelope and aids in viral particle formation, thereby facilitating virus assembly and release (4, 5). ANXA2 is also involved in the production processes of classical swine fever virus (CSFV) and hepatitis C virus (HCV) by binding to the NS5A protein (6). ANXA2 can induce the formation of lipid raft microdomains, thereby promoting viral assembly by recruiting the HCV replication complex (7). Furthermore, ANXA2 interacts with viral-encoded proteins from avian influenza virus (AIV) and porcine reproductive and respiratory syndrome virus (PRRSV) to enhance virus replication (8, 9). Given that ANXA2 promotes the replication of a diverse array of unrelated RNA viruses, we hypothesized that it might exert a broad-spectrum, virus-independent effect by targeting a common host process—the innate antiviral immune response, particularly the production of type I interferons (IFNs).

Innate immunity is the first line of defense against pathogenic microorganism infections and plays key roles in regulating host antiviral responses to eliminate pathogens. Upon pathogen infection, pattern recognition receptors (PRRs) can recognize pathogen-associated molecular patterns, including viral DNA, viral RNA, and surface glycoproteins, to activate host antiviral responses (10). The known PRRs include Toll-like receptors (TLRs), retinoic acid-inducible gene I (RIG-I)-like receptors (RLRs), nucleotide-binding domain and leucine-rich repeat-containing receptors, C-type lectin receptors, and PYRIN family members (11).

RIG-I and MDA5 are well-known RLRs that recognize viral RNAs in the process of RNA virus infections (12). Although RIG-I and MDA5 have distinct roles in recognizing different features of viral RNAs, both utilize the same downstream adaptor molecule, MAVS (13) (also known as IPS-1, VISA, or Cardif) (12, 14–16). Upon RNA virus infection, the carboxy-terminal domain of RIG-I/MDA5 interacts with viral RNA, inducing conformational changes that lead to their oligomerization (12) and exposure of their caspase activation recruitment domain (CARD). RIG-I/MDA5 CARD interacts with the CARD domain of MAVS, resulting in the recruitment of TRAFs to the linker between the CARD and transmembrane domain (TM) of MAVS (17). This recruitment activates TBK1 and IKKε (18), resulting in the phosphorylation and translocation of IRF3 to the nucleus to induce the production of type I IFN (19).

While previous studies have established that ANXA2 promotes the replication of diverse RNA viruses through direct interactions with specific viral proteins—such as the NS1 protein of AIV, the NSP9 protein of PRRSV, and the M protein of measles virus—these virus-specific mechanisms do not preclude the existence of a broader, common host pathway through which ANXA2 may exert a more universal pro-viral effect. Notably, the replication of numerous unrelated RNA viruses is potently restricted by the host type I IFN response. We therefore hypothesized that ANXA2, beyond its virus-specific roles, might function as a broad-spectrum modulator of host antiviral immunity by suppressing the production of type I IFNs, thereby creating a cellular environment conducive to the replication of a wide range of RNA viruses. This study was designed to test this hypothesis and to elucidate the underlying molecular mechanism. In this study, we identified ANXA2 as a negative regulator of type I IFN production by presenting biochemical and genetic evidence. ANXA2 inhibits the interactions of MDA5-MAVS and MAVS-TRAF3 during RNA virus infection, resulting in the suppression of type I IFN production and the facilitation of viral replication. In this study, we identified a novel and specific mechanism by which ANXA2 acts as a negative regulator of type I IFN production. We provide evidence that ANXA2 achieves this by directly targeting the RLR pathway, where it disrupts the critical protein-protein interactions between MDA5 and MAVS, and between MAVS and TRAF3.

## RESULTS

### ANXA2 inhibits type I IFN production

ANXA2 has been reported to regulate the replication of various RNA viruses, such as influenza A virus (IAV), HCV, PRRSV, and CSFV, through different mechanisms (20). Therefore, we propose a hypothesis on whether there is a broad-spectrum physiological function of ANXA2 to promote viral replication. We first investigated whether ANXA2 promotes viral replication by inhibiting the host’s innate immune response.

**Fig 1:**
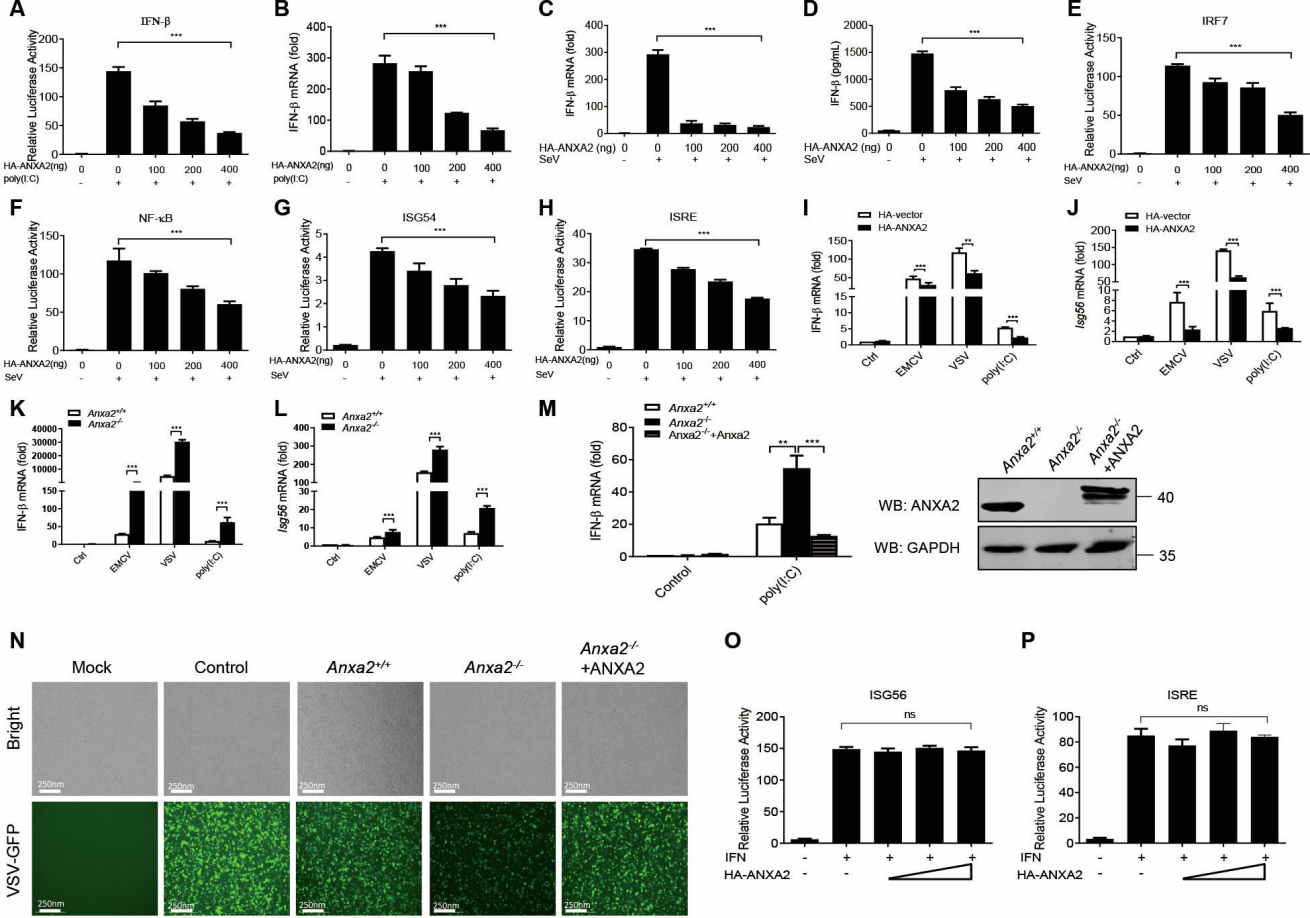
ANXA2 inhibits type I IFN production. (**A**) HEK293T cells were transfected with an IFN-β-Luc reporter and a Renilla-TK reporter, together with different amounts (0, 100, 200, and 400 ng) of a plasmid expressing HA-ANXA2 and then transfected with poly(I:C) (2 μg/mL). (**B**) qPCR analysis of the mRNA levels of *Ifnβ1* in HEK293T cells transfected with increasing amounts of plasmids expressing HA-ANXA2 and then transfected with poly(I:C) (2 μg/mL). (**C**) HEK293T cells transfected with an IFN-β-Luc reporter and a Renilla-TK reporter, together with different amounts (0, 100, 200, and 400 ng) of a plasmid expressing HA-ANXA2 and then infected with SeV (1 multiplicity of infection [MOI]) for 12 h. (**D**) qPCR analysis of the mRNA levels of *Ifnβ1* in HEK293T cells transfected with increasing amounts of plasmids expressing HA-ANXA2 and then infected with SeV (1 MOI) for 12 h. (**E–H**) HEK293T cells transfected with an IRF7- (**E**), ISG54- (**F**), ISRE- (**G**), or NF-κB-Luc reporter (**H**) and a Renilla-TK reporter, together with different amounts (0, 100, 200, and 400 ng) of a plasmid expressing HA-ANXA2 and then infected with SeV for 12 h. The cells were collected, and the Luc activities were detected. (**I and J**) qPCR analysis of the mRNA levels of *Ifnβ1* (**I**) and *Isg56* (**J**) in the HEK293T cells transfected with HA-vector or HA-ANXA2 and infected with EMCV or VSV or transfected with poly(I:C) (2 μg/mL) for 24 h. (**K and L**) qPCR analysis of the mRNA levels of *Ifnβ1* (**G**) and *Isg56* (**H**) in HEK293T-*Anxa2^+/+^* and HEK293T-*Anxa2^-/-^* cells after infection with EMCV or VSV for 12 h or transfection with poly(I:C) for 24 h. (**M**) qPCR analysis of the mRNA levels of *Ifnβ1* in the HEK293T-*Anxa2^+/+^* cells, HEK293T-*Anxa2^-/-^* cells, and HEK293T-*Anxa2^-/-^* cells transfected with a plasmid expressing HA-ANXA2, with or without EMCV infection for 12 h. Immunoblot analysis of ANXA2 expression in the HEK293T-*Anxa2^+/+^* cells, HEK293T-*Anxa2^-/-^* cells, and HEK293T-*Anxa2^-/-^* cells transfected with a plasmid expressing HA-ANXA2. (**N**) HEK293T-*Anxa2^+/+^* cells and HEK293T-*Anxa2^-/-^* cells transfected with an empty vector or a plasmid expressing ANXA2 were infected with SeV for 12 h. The cell supernatants were placed under ultraviolet (UV) aseptic irradiation for 12 h and then added to HEK293T cells and incubated for 24 h, and the HEK293T cells were infected with VSV-GFP. After 12 h, the replication of VSV-GFP was analyzed using a fluorescence microscope. (**O, P**) HEK293T cells transfected with an ISG56- (**O**), ISRE-Luc reporter (**P**) and a Renilla-TK reporter, together with different amounts (0, 100, 200, and 400 ng) of a plasmid expressing HA-ANXA2 and then stimulated with IFN for 12 h. The cells were collected, and the Luc activities were detected. ns, not significant (*P* > 0.05), *P < 0.05, **P < 0.01, and ***P < 0.001 (one-way ANOVA followed by Bonferroni post-test). Data are representative of three independent experiments with three biological replicates (the mean ± standard deviation [SD] of triplicate assays [A–P]) or are representative of three independent experiments with similar results.

To investigate the function of ANXA2 in host antiviral responses, we first tested whether ANXA2 inhibits IFN production. In human HEK293T cells, we found that overexpression of ANXA2 significantly inhibited the promoter activity of IFN-β and the mRNA levels of IFN-β induced by transfection with poly(I:C) and infection with Sendai virus (SeV) (Fig. 1A through D). ANXA2 also inhibited the activation of the IRF7, ISG54, ISRE, and NF-κB promoters induced by SeV in a dose-dependent manner (Fig. 1E through H). Similarly, the inhibitory effects of ectopically expressed ANXA2 on the mRNA levels of IFN-β and Isg56 were observed in HEK293T cells transfected with poly(I:C) or infected with other RNA viruses, such as encephalomyocarditis virus (EMCV) and vesicular stomatitis virus (VSV) (Fig. 1I and J). To further confirm these results, HEK293T cells with ANXA2 gene deletion (HEK293T-*Anxa2^-/-^*) were generated using CRISPR/Cas9 to confirm the function of ANXA2. As shown in Fig. 1K and L, ANXA2 deficiency markedly increased the mRNA levels of *IFN-β* and *Isg56* induced by infection with EMCV, VSV, or transfection with poly(I:C). Notably, reintroduction of ANXA2 into HEK293T-*Anxa2^-/-^* cells significantly attenuated the enhanced IFN-β mRNA expression caused by ANXA2 deficiency (Fig. 1M). An IFN sensitivity assay showed that the replication levels of VSV-GFP were correlated with the expression level of ANXA2 (Fig. 1N).

To rule out the potential effect of ANXA2 on the JAK-STAT signaling pathway, HEK293T cells were transfected with ANXA2-expressing plasmids and then treated with IFN. We found that the overexpression of ANXA2 did not affect the IFN-induced promoter activities of ISG56 and ISRE in the IFN signaling pathway (Fig. 1O and P). Taken together, our findings demonstrate that ANXA2 inhibits type I IFN production *in vitro*.

### ANXA2 deficiency enhances cellular antiviral responses *in vitro*

**Fig 2:**
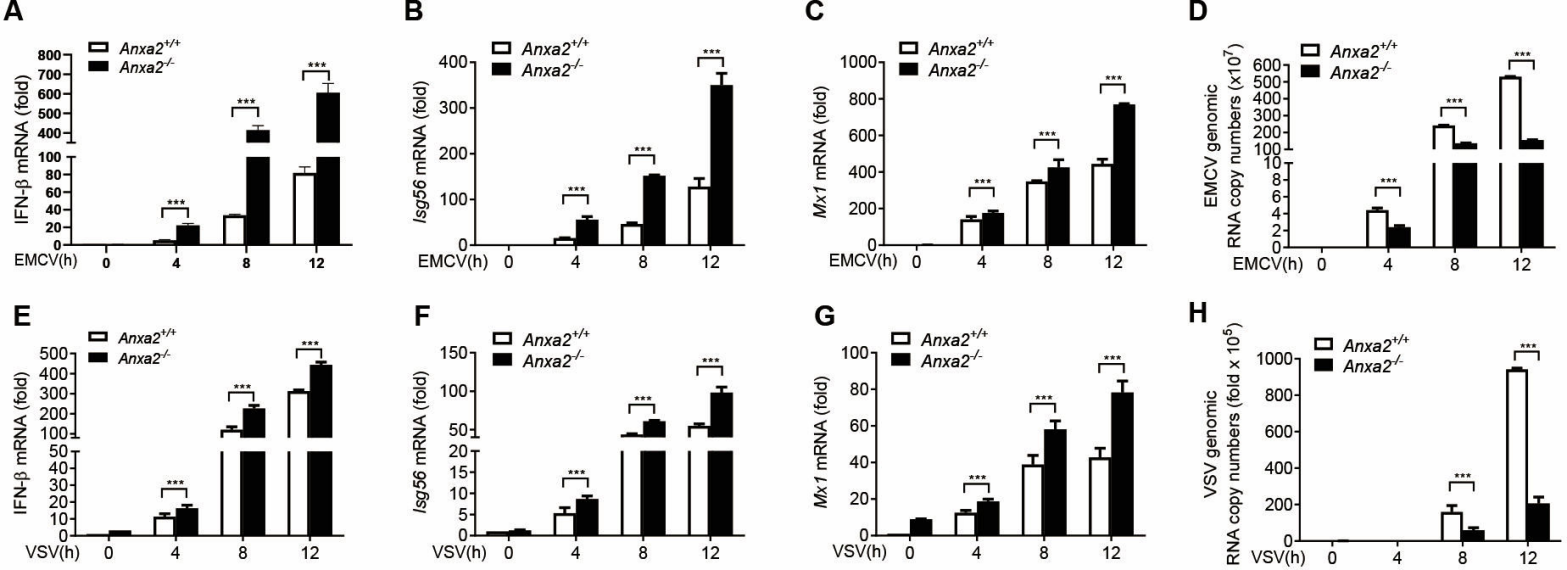
ANXA2 deficiency enhances cellular antiviral responses *in vitro*. (**A–C**) qPCR analysis of *Ifnb1* (**A**), *Isg56* (**B**), *Mx1* (**C**) mRNA levels in HEK293T*-Anxa2^+/+^* and HEK293T*-Anxa2^-/-^* cells infected with EMCV (2 MOI) for 0, 4, 8, or 12 h, respectively. (**D**) qPCR analysis of the genomic copy numbers of EMCV in HEK293T*-Anxa2^+/+^* and HEK293T*-Anxa2^-/-^* cells infected with EMCV for 0, 4, 8, or 12 h. (**E–G**) qPCR analysis of *Ifnb1* (**E**), *Isg56* (**F**), and *Mx1* (**G**) mRNA levels in HEK293T*-Anxa2^+/+^* and HEK293T*-Anxa2^-/-^* cells infected with VSV for 0, 4, 8, or 12 h. (**H**) qPCR analysis of the genomic copy numbers of VSV in HEK293T*-Anxa2^+/+^* and HEK293T*-Anxa2^-/-^* cells infected with VSV for 0, 4, 8, or 12 h. *P < 0.05, **P < 0.01, and ***P < 0.001 (two-tailed Student’s *t*-test). Data are representative of three independent experiments with three biological replicates (mean ± SD [**A–H**]) or are representative of three independent experiments with similar results.

To further validate the function of IFN inhibition by ANXA2 and its effect on viral replication, we infected cells using different RNA viruses. It was found that the mRNA levels of *IFN-β*, *Isg56,* and *Mx1* in the HEK293T-*Anxa2^-/-^* cells infected with EMCV and VSV were significantly higher than in wild-type cells after infection with EMCV (Fig. 2A through C) or VSV (Fig. 2E through G). Moreover, the EMCV and VSV genomic RNA copy numbers were significantly lower in the HEK293T-*Anxa2^-/-^* cells than in the HEK293T-*Anxa2^+/+^* cells (Fig. 2D and H). Collectively, these results indicate that ANXA2 deficiency enhances the type I IFN production as well as inhibits viral replication.

### ANXA2 inhibits type I IFN production upstream of IRF3 phosphorylation

**Fig 3:**
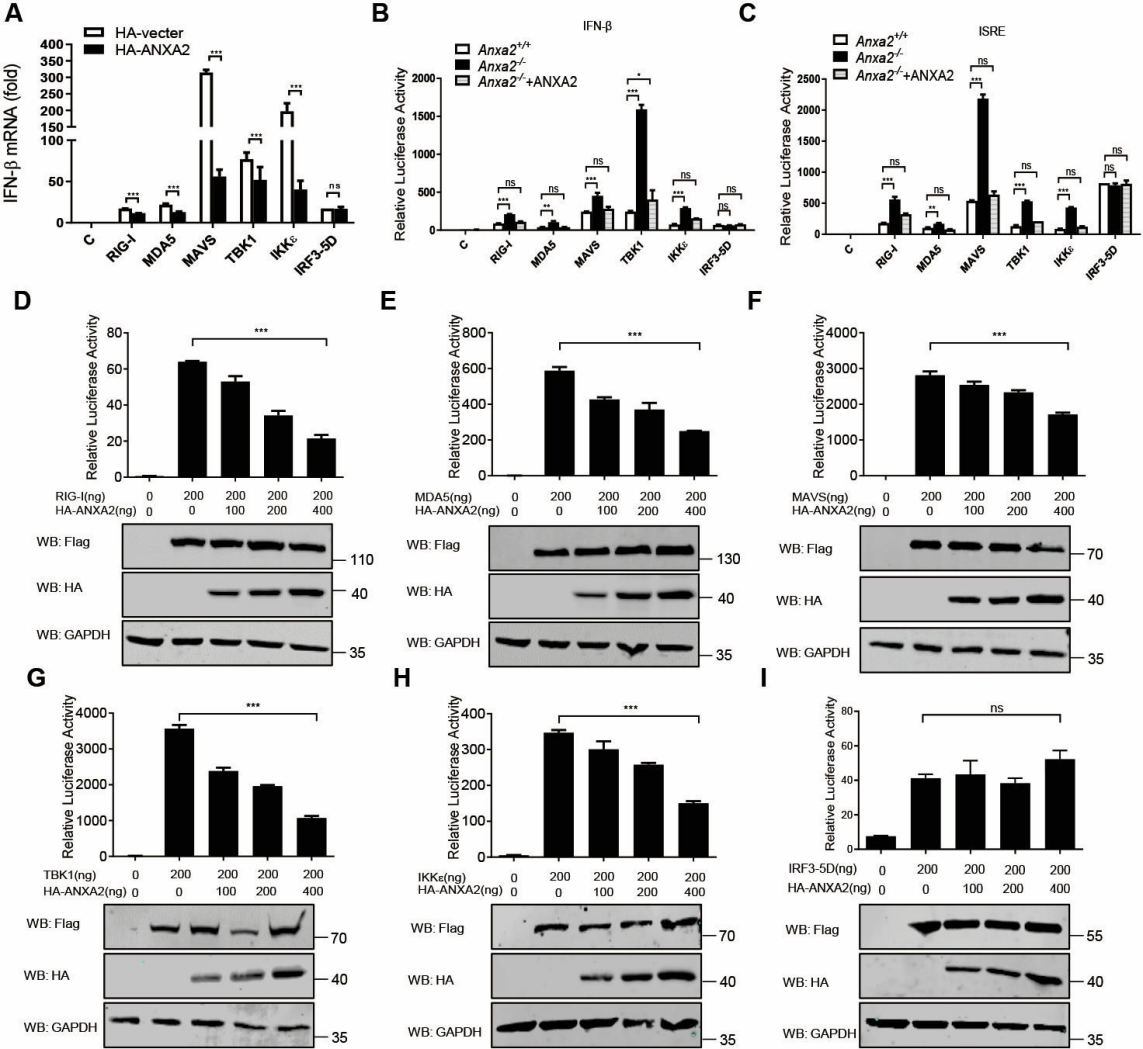
ANXA2 inhibits type I IFN production upstream of IRF3 phosphorylation. (**A**) qPCR analysis of the mRNA levels of *Ifnβ1* in the HEK293T cells transfected with a plasmid expressing RIG-I, MDA-5, MAVS, TBK1, IKKε, or IRF3-5D, along with an empty vector or a plasmid expressing ANXA2. (**B and C**) Luc activity of the IFN-β-Luc (**B**) or ISRE-Luc (**C**) reporter in the HEK293T-*Anxa2^+/+^* and HEK293T-*Anxa2^-/-^* cells transfected with an IFN-β-Luc or ISRE-Luc reporter and a Renilla-TK reporter together with a plasmid expressing RIG-I, MDA-5, MAVS, TBK1, IKKε, or IRF3-5D, along with an empty vector or a plasmid expressing ANXA2. (**D–I**) Luc activity of the IFN-β promoter reporter in HEK293T cells transfected with an IFN-β Luc reporter and a Renilla-TK reporter, together with a plasmid expressing RIG-I, MDA-5, MAVS, TBK1, IKKε, or IRF3-5D along with empty vector or increasing amounts of a plasmid expressing ANXA2. The expressions of RIG-I, MDA5, MAVS, TBK1, IKKε, ANXA2, and GAPDH were detected by Western blotting. ns, not significant (*P* > 0.05), *P < 0.05, **P < 0.01, and ***P < 0.001 (two-tailed Student’s *t*-test [**A–I**]). Data are representative of three independent experiments with three biological replicates (mean ± SD [**A–C**]) or are representative of three independent experiments with similar results (**D–I**).

To elucidate the underlying molecular mechanisms by which ANXA2 negatively regulates type I IFN production in HEK293T cells, we first assessed the effect of ANXA2 on IFN-β promoter activation induced by key molecules in the type I IFN signaling pathway. HEK293T cells were transfected with a plasmid expressing RIG-I, MDA5, cGAS+STING, MAVS, TBK1, or IKKε, along with HA-ANXA2. qPCR results showed that the ectopic expression of ANXA2 reduced the mRNA levels of IFN-β induced by the indicated molecules, but not by IRF3-5D (Fig. 3A). Subsequently, HEK293T-*Anxa2^+/+^* and HEK293T-*Anxa2^-/-^* cells were transfected with a plasmid expressing RIG-I, MDA5, cGAS+STING, MAVS, TBK1, or IKKε, along with IFN-β and ISRE promoter reporters. We found that the IFN-β and ISRE promoter activities induced by RIG-I, MDA5, cGAS+STING, MAVS, TBK1, and IKKε were significantly increased in HEK293T*-Anxa2^-/-^* cells compared to HEK293T-*Anxa2^+/+^* cells. Additionally, overexpression of ANXA2 in HEK293T-*Anxa2^-/-^* cells restored the inhibitory effects of ANXA2 on RIG-I-, MDA5-, cGAS+STING-, MAVS-, TBK1-, and IKKε-mediated IFN-β and ISRE promoter activities, but not IRF3-5D (Fig. 3B through C). Consistent with these results, ectopically expressed ANXA2 significantly decreased the IFN-β promoter activation induced by RIG-I, MDA5, cGAS+STING, MAVS, TBK1, or IKKε in a dose-dependent manner, while the activation of the IFN-β promoter induced by IRF3-5D (a constitutively active IRF3) was not affected (Fig. 3D through I). These results suggest that ANXA2 may inhibit type I IFN production upstream of IRF3 phosphorylation.

### ANXA2 inhibits the interaction of MDA5-MAVS during RNA virus infection

**Fig 4:**
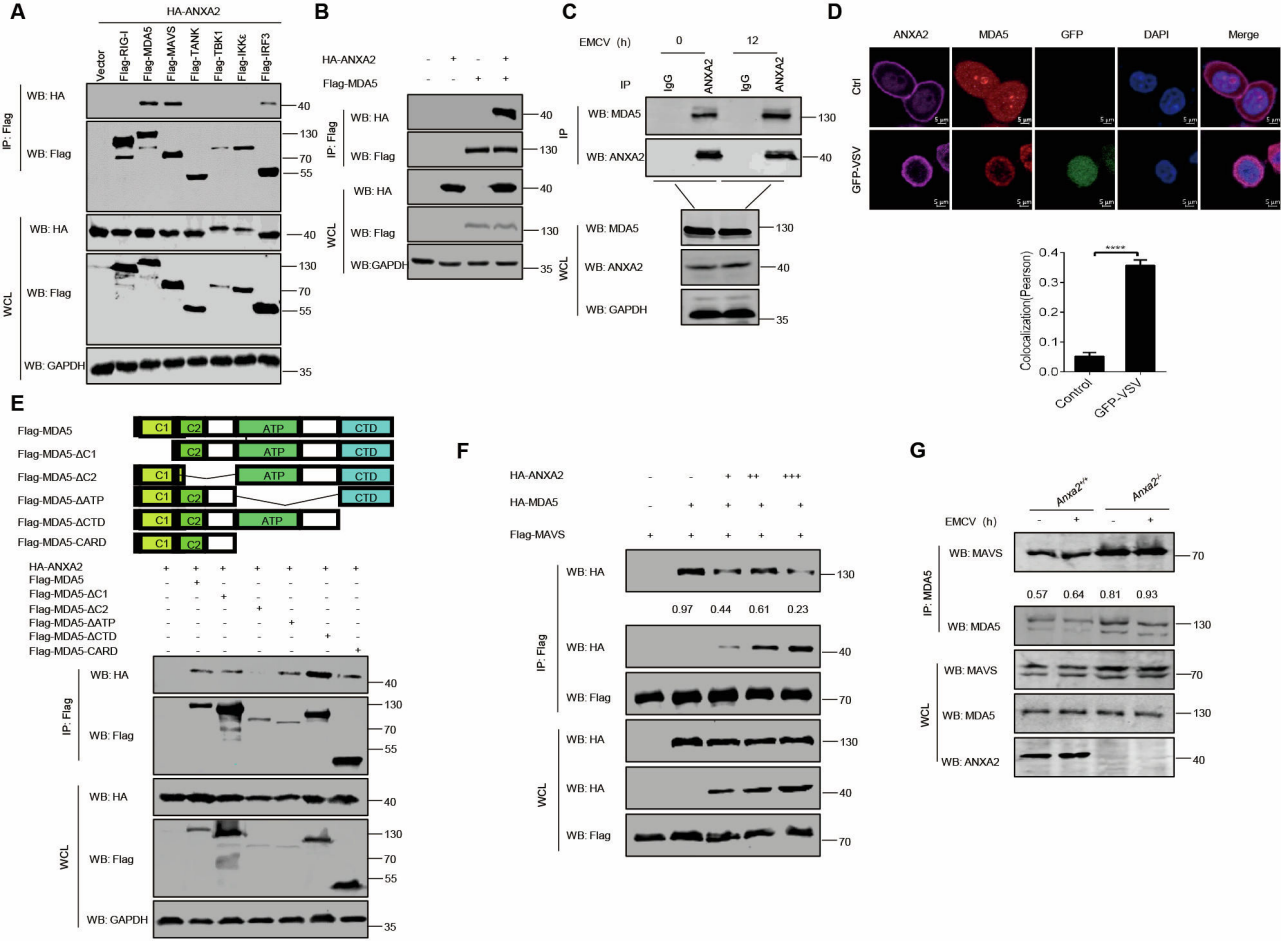
ANXA2 inhibits the recruitment of MAVS by MDA5. (**A**) Co-IP analysis was performed to detect the interaction between ANXA2 and immune molecules in HEK293T cells. HEK293T cells were transfected with a plasmid expressing HA-ANXA2 and a plasmid expressing Flag-tagged RIG-I, MDA-5, MAVS, TANK, TBK1, IKKε, and IRF3, respectively. (**B**) Co-IP analysis of the interaction between ANXA2 and MDA5 in HEK293T cells transfected with plasmids expressing HA-ANXA2 and Flag-MDA5. (**C**) Co-IP analysis of the interaction between endogenous ANXA2 and MDA5 in mouse peritoneal macrophages that were either mock-infected or infected with EMCV. (**D**) HEK293T cells were mock-infected or infected with GFP-VSV (0.1 MOI) for 12 h. The subcellular localization of endogenous ANXA2 and MDA5 was analyzed by fluorescence microscopy. The Pearson’s correlation coefficient and Mander’s overlap coefficient of the images were analyzed using the Zeiss processing system. (**E**) MDA5 and its truncation mutants (above). Co-IP analysis of the interaction between ANXA2 and MDA5 or its deleted mutants in HEK293T cells was transfected with a plasmid expressing HA-ANXA2 together with vector or Flag-MDA5 and its deleted mutants (below). (**F**) Co-IP analysis of the interactions among ANXA2, MDA5, and MAVS in HEK293T cells transfected with plasmids expressing Flag-MAVS together with HA-MDA5 and vector or increasing amounts of a plasmid encoding HA-ANXA2. (**G**) Co-IP analysis of the interaction between endogenous MDA5 and MAVS in mouse peritoneal macrophages that were either mock-infected or infected with EMCV. Data represented are from three independent experiments with three biological replicates (mean ± SD) or from three independent experiments with consistent results (**A–G**).

To identify the target of ANXA2, we examined the interaction between ANXA2 and key molecules involved in RLR signaling pathways. As shown in Fig. 4A, ANXA2 interacted with MDA5, MAVS, and IRF3 when these proteins were co-expressed with ANXA2 in HEK293T cells. The interaction between ANXA2 and MDA5 was validated (Fig. 4B). Consistent with this result, endogenous MDA5 also interacted with endogenous ANXA2 regardless of mock infection or infection with EMCV (Fig. 4C). Additionally, IFA results demonstrated the co-localization of ANXA2 with MDA5 at the cell membrane during GFP-VSV infection (Fig. 4D).

Five truncated mutants of MDA5 (MDA5-ΔC1, MDA5-ΔC2, MDA5-ΔATP, MDA5-ΔCTD, and MDA5-CARD) were constructed to identify the domain of MDA5 necessary for its interaction with ANXA2. As shown in Fig. 4E, ANXA2 interacted with MDA5-WT, MDA5-ΔC1, MDA5-ΔATP, MDA5-ΔCTD, and MDA5-CARD but not with MDA5-ΔC2, indicating that the CARD domain of MDA5 is required for its interaction with ANXA2.

Upon EMCV infection, MDA5 senses EMCV genomic RNA and then recruits MAVS through its CARD domain (21) to activate TBK1. We proposed that ANXA2 may inhibit this interaction. Co-IP results revealed that ANXA2 inhibited the interaction between MDA5 and MAVS (Fig. 4F). Consistent with this result, the interaction between MDA5 and MAVS increased in HEK293T-*Anxa2^-/-^* cells compared with HEK293T-*Anxa2*^+/+^ cells upon EMCV infection (Fig. 4G).

### ANXA2 disrupts the MAVS-TRAF3 interaction

**Fig 5:**
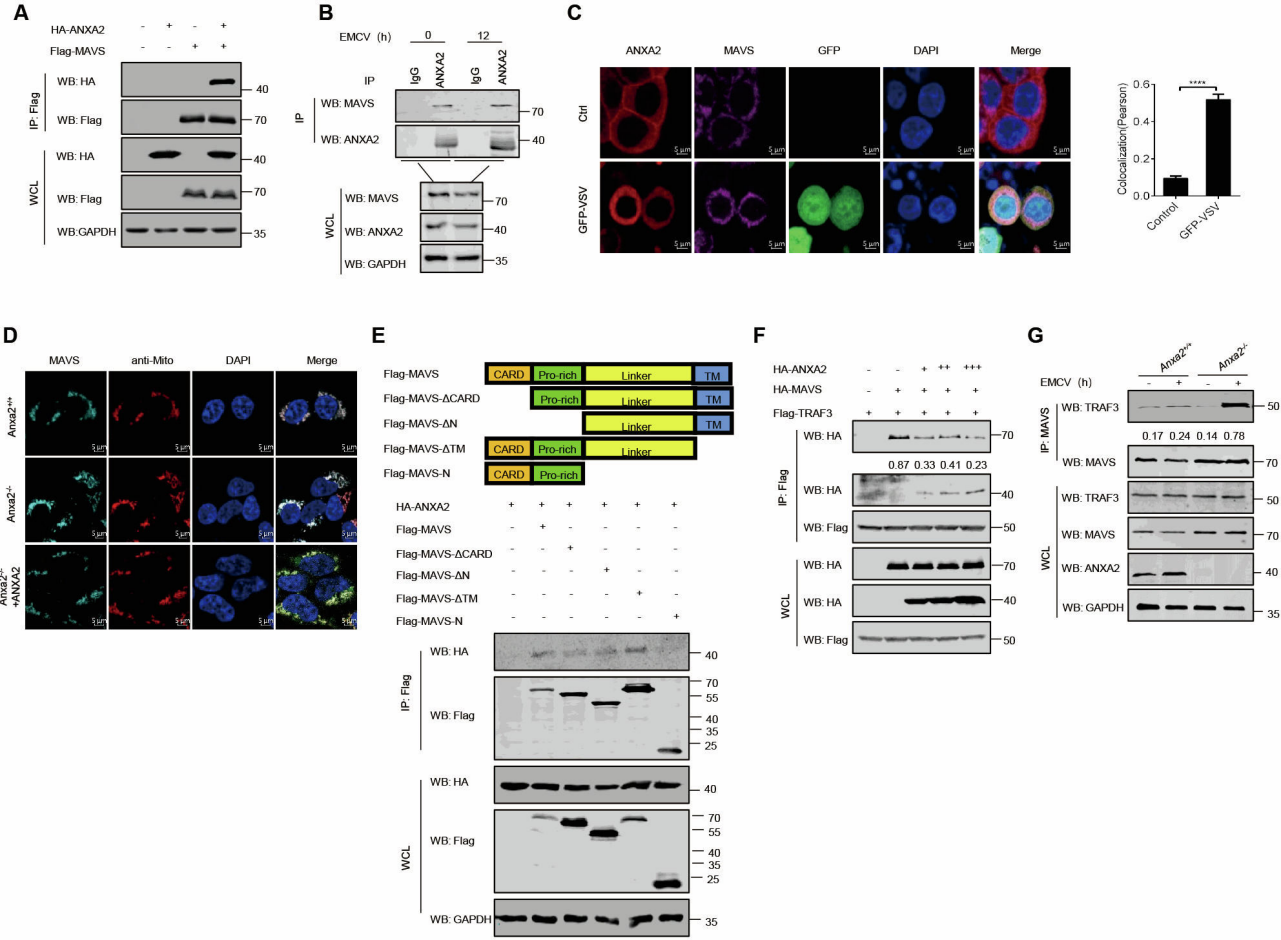
ANXA2 disrupts the interaction between MAVS and TRAF3. (**A**) Co-IP analysis of the interaction between ANXA2 and MAVS in HEK293T cells transfected with plasmids expressing HA-ANXA2 and Flag-MAVS. (**B**) Co-IP analysis of the interaction between endogenous ANXA2 and MAVS in mouse peritoneal macrophages that were either mock-infected or infected with EMCV. (**C**) HEK293T cells were mock-infected or infected with GFP-VSV (0.1 MOI) for 12 h. The subcellular localization of endogenous ANXA2 and MDA5 was analyzed by fluorescence microscopy. The Pearson’s correlation coefficient and Mander’s overlap coefficient of the images were analyzed using the Zeiss processing system. (**D**) The subcellular localization of MAVS in HEK293T-*Anxa2^+/+^* and HEK293T-*Anxa2^-/-^* cells was detected by immunofluorescence microscopy. Scale bars, 5 μm. (**E**) MAVS and its truncation mutants (above). Co-IP analysis of the interaction between ANXA2 and MAVS or its deleted mutants in HEK293T cells was transfected with a plasmid expressing HA-ANXA2 together with vector or Flag-MAVS and its deleted mutants (below). (**F**) Co-IP analysis of the interactions among ANXA2, TRAF3, and MAVS in HEK293T cells transfected with plasmids expressing Flag-MAVS together with HA-TRAF3 and vector or increasing amounts of a plasmid encoding HA-ANXA2. (**G**) Co-IP analysis of the interaction between endogenous MAVS and TRAF3 in mouse peritoneal macrophages that were either mock-infected or infected with EMCV. Data represent three independent experiments with three biological replicates (mean ± SD) or represent three independent experiments with similar results (**A–G**).

Co-IP results also demonstrated that ectopically expressed ANXA2 interacted with MAVS (Fig. 5A). Additionally, the interaction between endogenous ANXA2 and MAVS in mock-infected or EMCV-infected HEK293T cells was confirmed (Fig. 5B). IFA results revealed that ANXA2 colocalized with MAVS in the cytoplasm (Fig. 5C), and the localization of MAVS on the mitochondria was not affected by the overexpression or deletion of ANXA2 (Fig. 5D).

Similarly, four deletion mutants of MAVS were generated to identify its binding domain to ANXA2 (Fig. 5E). As shown in Fig. 5E, MAVS-WT, MAVS-ΔCARD, MAVS-ΔN, and MAVS-ΔTM interacted with ANXA2, but not MAVS-N, indicating that ANXA2 interaction with MAVS is dependent on the linker between the CARD and TM domains of MAVS. Previous studies have shown that the linker between CARD and TM of MAVS is necessary for its recruitment of TRAFs (22). To test whether ANXA2 has an effect on the interaction between MAVS and TRAF3, HEK293T cells were transfected with plasmids expressing Flag-MAVS and HA-TRAF3, along with an increasing amount of a plasmid expressing GFP-ANXA2. As shown in Fig. 5F, ANXA2 inhibited the interaction between MAVS and TRAF3 in a dose-dependent manner. Consistent with the above results, the interaction between MAVS and TRAF3 was enhanced in HEK293T-*Anxa2^-/-^* cells compared to that in the HEK293T-*Anxa2^+/+^* cells upon EMCV infection (Fig. 5G).

### ANXA2 deficiency enhances host antiviral responses *in vivo*

**Fig 6:**
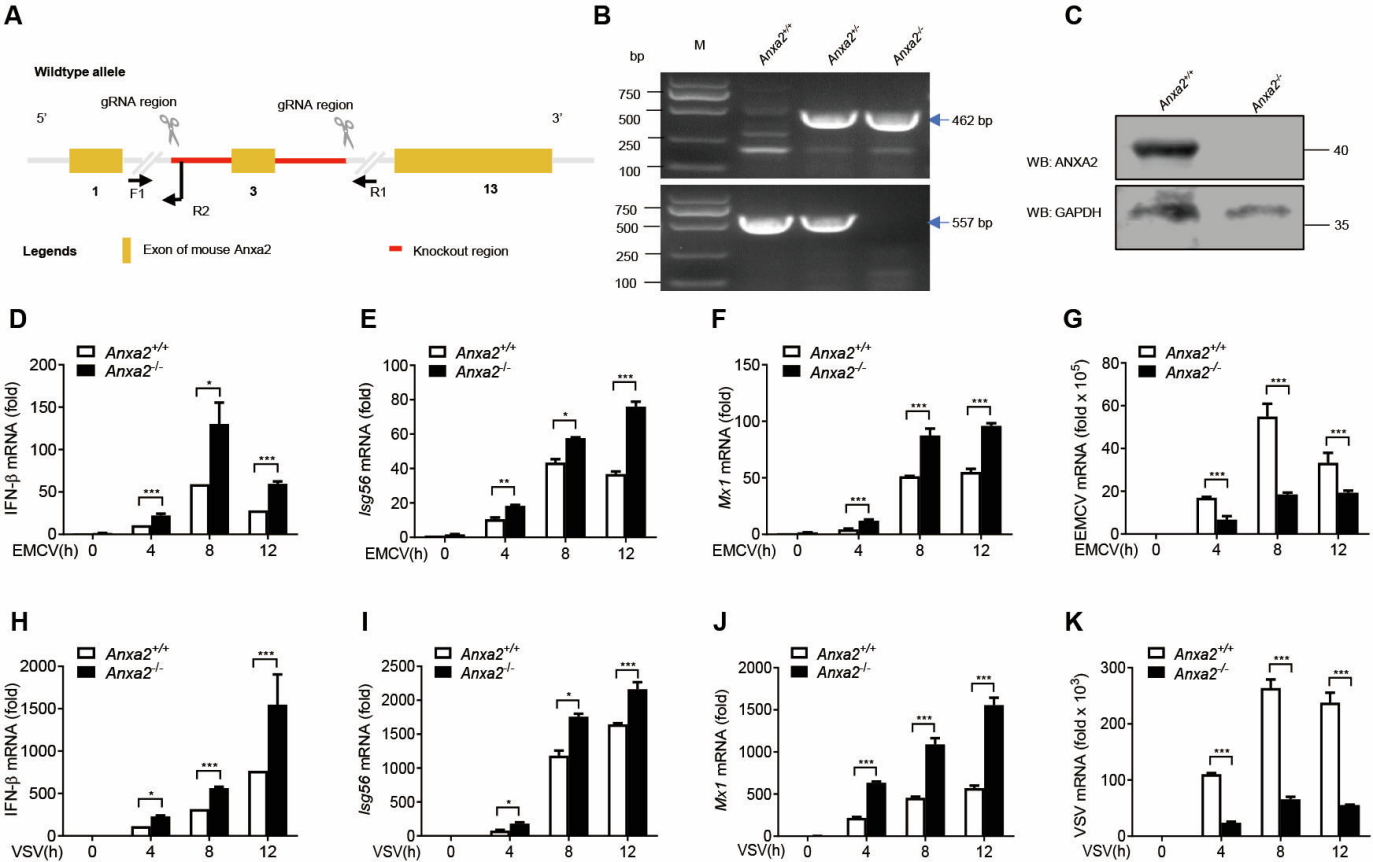
ANXA2 deficiency promotes type I IFN production and thus inhibits viral replication. (**A**) Schematic diagram of the homologous recombination-mediated Anxa2 gene knockout strategy in mice. (**B**) Immunoblot analysis of ANXA2 protein expression in *Anxa2^+/+^* and *Anxa2^-/-^* peritoneal macrophages. (**C**) The genotypes and phenotypes of the *Anxa2*^-/-^ mice were analyzed. (**D–F**) qPCR analysis of the mRNA levels of *Ifnβ1* (**D**), *Isg56* (**E**), and *Mx1* (**F**) in *Anxa2^+/+^* and *Anxa2^-/-^* peritoneal macrophages infected with EMCV (2 MOI) for 0, 4, 8, or 12 h. (**G**) qPCR analysis of the genomic copy numbers of EMCV in the peritoneal macrophages isolated from *Anxa2^+/+^* and *Anxa2^-/-^* mice infected with EMCV for 0, 4, 8, or 12 h. (**H–J**) qPCR analysis of the mRNA levels of *Ifnβ1* (**H**), *Isg56* (**I**), and *Mx1* (**J**) in *Anxa2^+/+^* and *Anxa2^-/-^* peritoneal macrophages infected with VSV (0.5 MOI) for 0, 4, 8, or 12 h. (**K**) qPCR analysis of the genomic copy numbers of VSV in the peritoneal macrophages isolated from *Anxa2^+/+^* and *Anxa2^-/-^* mice infected with VSV for 0, 4, 8, or 12 h. *P < 0.05, **P < 0.01, and ***P < 0.001 (two-tailed Student’s *t*-test (**D–K**). Data are representative of three independent experiments with three biological replicates (mean ± SD [**D–K**]).

To investigate whether ANXA2 regulates type I IFN production *in vivo*, *Anxa2* knockout (*Anxa2^-/-^*) mice were generated using a homologous recombination technique and confirmed by sequencing and Western blot analysis (Fig. 6A through C). Primary peritoneal macrophages isolated from *Anxa2^-/-^* mice and their wild-type (WT) littermates (*Anxa2^+/+^*) were infected with EMCV and VSV for 0, 4, 8, and 12 h. Compared to macrophages from *Anxa2^+/+^* mice, macrophages from *Anxa2^-/-^* mice had higher mRNA expression of IFN-β (Fig. 6D and 2H), *Isg56* (Fig. 6E and I), and *Mx1* (Fig. 6F and J) at different time points. Furthermore, the EMCV and VSV genomic RNA copy numbers in the macrophages from *Anxa2^-/-^* mice were significantly lower than that of the macrophages from *Anxa2^+/+^* mice at 0, 4, 8, and 12 hpi (Fig. 6G and K). Collectively, these results indicate that ANXA2 deficiency enhances type I IFN production and inhibits viral replication.

**Fig 7:**
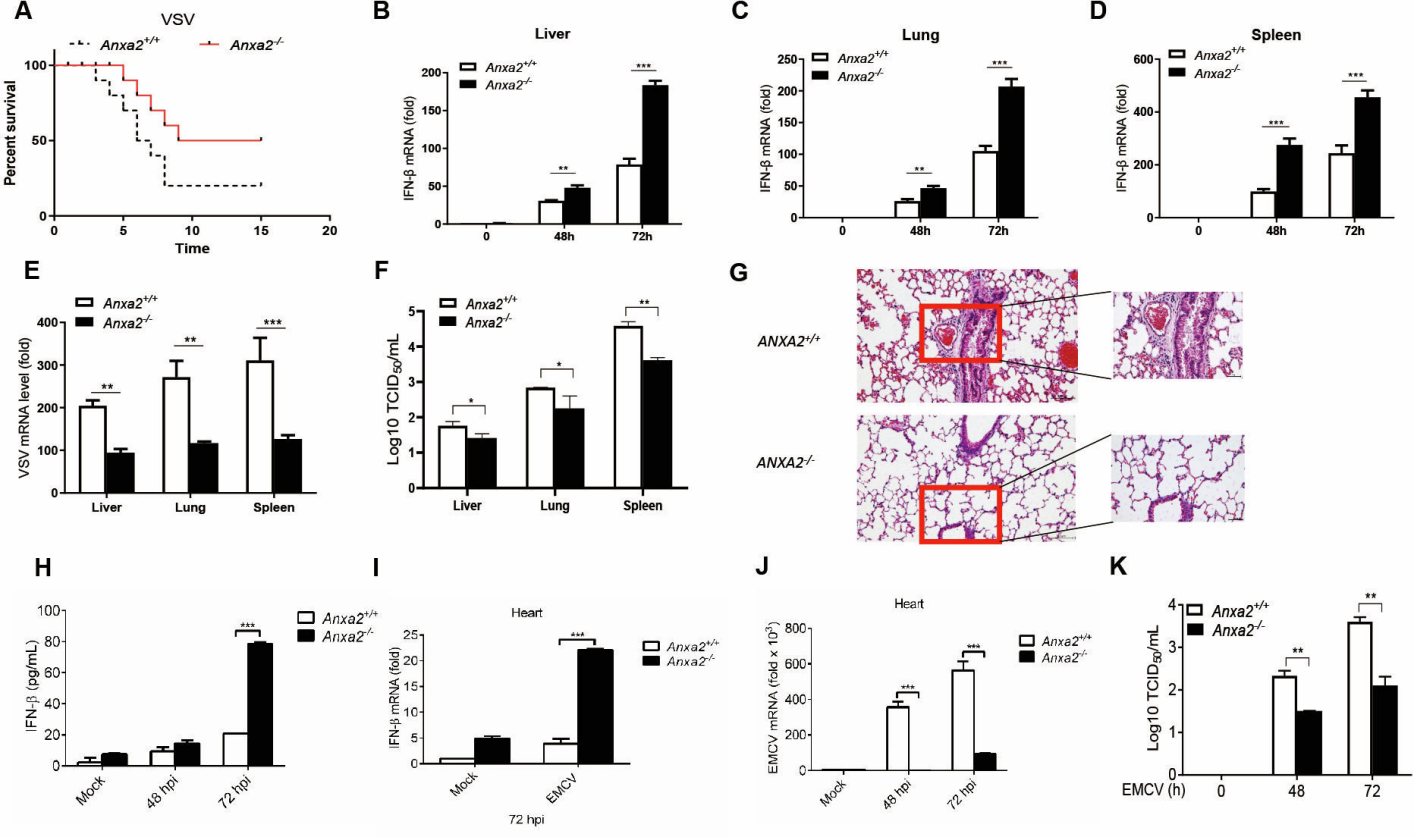
ANXA2 deficiency positively regulates antiviral responses *in vivo*. (**A**) *Anxa2^+/+^* and *Anxa2^-/-^* mice (10 mice per group) were intraperitoneally injected with VSV (1 × 10^4.5^ PFU per mouse), and the survival ratio of these mice was observed daily. (**B–D**) The mRNA levels of *Ifnb1* in the liver (**B**), lung (**C**), and spleen (**D**) isolated from these mice as in panel **A** were analyzed by qPCR at 48 hpi and 72 hpi; mRNA results are presented relative to those in the uninfected WT mice. The genomic copy numbers of VSV in the liver, lung, and spleen isolated from these mice, as in panel **A**, were analyzed by qPCR at 72 hpi. (**F**) The TCID_50_ assay was used to test VSV titer in the liver, lung, and spleen of mice as described in panel **A**. (**G**) Hematoxylin and eosin-stained images of lung sections from *Anxa2^+/+^* and *Anxa2*^-/-^ mice infected with VSV for 72h. Scale bars, 50 μm. (**H**) The *Anxa2*^+/+^ and *Anxa2*^-/-^ mice (four mice per group) were infected by intraperitoneal injection of EMCV (2 × 10^5^ PFU per mouse) for 48 h and 72 h. Detection of IFN-β levels in serum from mice by ELISA. (**I**) The mRNA levels of *Ifnβ1* in the heart were analyzed by qPCR analysis. (**J**) qPCR analysis of EMCV RNA in the hearts of *Anxa2*^+/+^ and *Anxa2*^-/-^ mice as described in A. (**K**) The TCID_50_ assay was used to test EMCV titer in the hearts of mice, as described in panel **A**. *P < 0.05, **P < 0.01, and ***P < 0.001 (two-tailed Student’s *t*-test [**A–K**]). Data are representative of three independent experiments with three biological replicates (mean ± SD [**A–J**]) or are representative of three independent experiments with similar results (**A–K**).

To further define the function of ANXA2 in inhibiting type I IFN production and host antiviral responses *in vivo*, *Anxa2^-/-^* mice and *Anxa2^+/+^* mice were challenged with VSV via intraperitoneal injections. As shown in Fig. 7A, *Anxa2^-/-^* mice were more resistant to VSV infection than *Anxa2^+/+^* mice. Furthermore, we found that the mRNA levels of *Ifnβ1* in the liver, lung, and spleen from *Anxa2^-/-^* mice were significantly higher than those from *Anxa2^+/+^* mice after infection with VSV for 48 h and 72 h (Fig. 7B through D). Additionally, the VSV viral genomic RNA copies in the liver, lung, and spleen were significantly lower in *Anxa2^-/-^* mice than in *Anxa2^+/+^* mice (Fig. 7E). The VSV titers in the liver, lung, and spleen from the *Anxa2^-/-^* mice were significantly lower than those in *Anxa2^+/+^* mice after infection with VSV for 72 h (Fig. 7F). Fewer signs of severe inflammation and less pathologic damage were observed in the lung tissue of *Anxa2-/-* mice compared with WT mice (Fig. 7G).

Using EMCV as a model, we infected *Anxa2^-/-^* mice and *Anxa2^+/+^* mice by injecting them intraperitoneally with EMCV, and we obtained similar results. Correspondingly, the protein levels of IFN-β in the serum from *Anxa2^-/-^* mice were significantly increased (Fig. 7H), while the mRNA levels of *Ifnβ1* in the heart from *Anxa2^-/-^* mice were significantly higher than those from *Anxa2^+/+^* mice after infection with EMCV for 72 h (Fig. 7I). Consistent with these results, the EMCV genomic copy numbers in the heart from the *Anxa2^-/-^* mice were significantly lower than those in *Anxa2^+/+^* mice after infection with EMCV for 48 and 72 h (Fig. 7J). The EMCV titers in the hearts of *Anxa2^-/-^* mice were significantly lower than those in *Anxa2^+/+^* mice after infection with EMCV for 72 h (Fig. 7K).

Based on these data, ANXA2 deficiency enhances the host’s antiviral immune response, thereby inhibiting viral replication.

## DISCUSSION

Annexins are a family of evolutionarily conserved multifunctional proteins that are widely distributed in various tissues and cells of plants and animals. They are associated with apoptosis and tumorigenesis (23–25) and innate immune responses involved in type I IFN production and inflammatory responses (26, 27). Recent evidence has shown that annexins are important in regulating host antiviral responses. For instance, the C-terminus of ANXA1 directly interacts with TBK1 to enhance the TLR-mediated IFN-β production in the TLR4/TLR3-TRIF signaling pathway (28). ANXA1 also affects type I IFN production by promoting IFN-β production after RIG-I stimulation. Conversely, knockdown of ANXA1 expression delays the phosphorylation of IRF3 and STAT1, leading to lower expression of ISGs, such as IFIT1 (29). Another member of the annexin family, ANXA7, enhances the IFN-β promoter activity induced by chicken MDA5 (chMDA5), thereby inhibiting the infection of the recombinant H5N1 virus (rNS1-SD30) lacking the eIF4GI-binding domain of NS1 (30).

As a membrane scaffold and binding protein, ANXA2 is mainly expressed on the plasma membrane and intracellular vesicles (2, 3), where it participates in the replication process of a variety of viruses. For example, ANXA2 interacts with the non-structural protein 1 (NS1) of AIV to increase viral replication (8). Our previous studies have shown that ANXA2 interacts with the non-structural protein 9 (NSP9) of PRRSV to promote viral replication, and vimentin interacts with the N protein of PRRSV in the presence of ANXA2 (9). In this study, we found that the overexpression of ANXA2 inhibited RNA virus (SeV, VSV, or EMCV)-induced IFN-β production, whereas ANXA2 deficiency enhanced type I IFN production and suppressed virus replication both *in vitro* and *in vivo*. RIG-I-like helicases, such as RIG-I, MDA5, and LGP2, act as important cytosolic PRRs to sense viral dsRNA. RIG-I and MDA5 transduce antiviral signals by interacting with MAVS via the CARD domain. MAVS then recruits TRAF3 through the linker between CARD and TM to activate downstream kinases, such as TBK1 and IKKε, to phosphorylate IRF3, leading to increased production of type I IFN and expression of antiviral genes (18, 31). Our findings demonstrate that ANXA2 interacts with the CARD domain of MDA5 and the linker region of MAVS, concurrently disrupting both the MDA5-MAVS and MAVS-TRAF3 interactions. While the precise structural mechanism awaits further elucidation, we propose two plausible, non-mutually exclusive models for how ANXA2, acting as a scaffold protein, achieves this dual inhibition. First, ANXA2 may function through competitive binding: by occupying the CARD domain of MDA5, it could sterically hinder MDA5’s interaction with the CARD of MAVS; simultaneously, its binding to the MAVS linker region—a critical site for TRAF3 recruitment—could directly compete with TRAF3 for access. Second, ANXA2 might facilitate the formation of a non-productive ternary complex with MDA5 and MAVS. In this scenario, ANXA2 could nucleate an abortive signaling complex that locks MDA5 and MAVS in a conformation incompatible with the subsequent recruitment and activation of downstream effectors like TRAF3. Future structural studies, such as determining the crystal or cryo-EM structure of the ANXA2-MDA5-MAVS complex, will be essential to distinguish between these models and to define the exact molecular architecture of this inhibitory node.

Our discovery that ANXA2 dampens type I IFN production by disrupting RLR signaling provides a mechanistic explanation for its previously observed anti-inflammatory role (20, 32, 33). By suppressing an overzealous IFN response, ANXA2 may help prevent immunopathology. However, this homeostatic function is exploited by multiple RNA viruses to facilitate their replication. This dual role (suppressing excessive IFN responses and facilitating viral replication) underscores the delicate balance between effective antiviral defense and detrimental inflammation, positioning ANXA2 at a critical crossroads of host-pathogen interaction. The enhanced survival of *Anxa2^-/-^* mice upon VSV challenge highlights the potential therapeutic benefit of temporally inhibiting ANXA2 during acute viral infections.

In summary, we identified a novel function of ANXA2 involved in host antiviral responses. An intriguing aspect of our findings is that ANXA2 constitutively interacts with MAVS under steady-state conditions without suppressing basal IFN signaling or altering MAVS mitochondrial localization. This suggests that ANXA2 functions not as a simple “off switch” but as a signal-responsive brake on the RLR pathway. We propose a model of “infection-triggered enhancement of inhibition” to explain this dynamic regulation. In uninfected cells, the ANXA2-MAVS interaction may be relatively low affinity, involve a limited fraction of MAVS pools, or occur in a conformation that only partially obstructs signaling. Viral infection, however, likely activates ANXA2’s full inhibitory potential through several mechanisms: (i) enhanced recruitment: the oligomerization of MDA5 and the subsequent formation of large MAVS aggregates on mitochondrial membranes upon viral sensing may create high-avidity platforms that recruit and concentrate ANXA2, amplifying its local inhibitory effect; (ii) post-translational modification: infection-induced kinase cascades could phosphorylate ANXA2 or its binding partners, modulating their interaction affinities or functional outcomes; (iii) altered competition: viral infection might shift the balance between positive and negative regulators at the MAVS signalosome, favoring the dominance of ANXA2’s inhibitory function. This model positions ANXA2 as a latent regulatory factor that helps maintain immune homeostasis by preventing aberrant IFN activation, yet whose suppressive activity is precisely amplified during viral infection to fine-tune the immune response and potentially limit immunopathology. Elucidating the specific “triggering” molecular events represents a key direction for future research.

## MATERIALS AND METHODS

### Mice

*Anxa2^-/-^* mice generated by homologous recombination technology were purchased from Saiye Biotechnology Co., Ltd. (Guangzhou, China). The mouse genotype was confirmed by PCR using the following primers: forward 5′-CAACTGAGGCACACTCACAAGCG-3′, reverse 5′-GAGAAGGGCTGGCTTAGGGCACT-3′, and 5′-ACTGTGCTGTGAATGCCCACCTTG-3′. All mice were bred in specific pathogen-free (SPF) barrier facilities at the Harbin Veterinary Research Institute (HVRI) of the Chinese Academy of Agricultural Sciences in Harbin, China. . Male and female *Anxa2^-/-^* and wild-type littermates (6–8 weeks old) were used throughout the experiments.

### Cell lines

Human HEK293T cells were purchased from the American Type Culture Collection (Manassas, VA). Human HEK293T cells were cultured in Dulbecco’s Modified Eagle’s Medium supplemented with 10% fetal bovine serum (FBS), 100 U/mL penicillin, and 100 μg/mL streptomycin at 37°C with 5% CO_2_. Primary mouse peritoneal macrophages were isolated from mice 3 days after injection of thioglycollate (MERCK) and cultured in RPMI 1640 medium supplemented with 10% FBS, 100 U/mL penicillin, and 100 mg/mL streptomycin at 37°C with 5% CO_2_.

### Viruses

The Sendai/Cantell strain (SeV strain, product code VR-907) was purchased from the American Type Culture Collection (ATCC) and amplified in SPF embryonated chicken eggs. The EMCV HB10 strain was provided by HVRI, and the VSV strain and VSV strain expressing GFP (VSV-GFP) were kindly provided by Prof. Zhigao Bu (HVRI, China).

### Plasmids

Plasmids expressing Flag-tagged RIG-I, MDA5, MAVS, TANK, TBK1, IKKε, IRF3, and IRF3-5D were constructed and stored in our laboratory. The IFN-β reporter, ISRE reporter, and TK-Renilla reporter were obtained from Prof. Hong Tang. To construct plasmids expressing HA- or Flag-tagged ANXA2, cDNAs corresponding to the human ANXA2 gene (NCBI Reference Sequence: NM_001002857.2) were amplified by standard reverse transcription-polymerase chain reaction (RT-PCR) using total RNA extracted from HEK293T cells as templates and were then cloned into the pCAGGS-HA/Flag vector. All constructs were validated by DNA sequencing. The primers used in this study are available upon request (Table 1).

**TABLE 1:** Primers used for PCR in this study

Plasmid	Primers (5′−3′)
pCAGGS-ANXA2 (Flag, HA)	F: TGCGAATTCGAGCTCATCGATGGTACCATGTCTACTGTTCACG R: TTAATTAATTAAGATCTGCTAGCTCGAGTCAGTCATCTCCACCA
pCAGGS-Flag-MDA5-ΔC1	F: GAATTCGAGCTCATCGATGGTACCGCTCATGATGAATATCTC R: TTAATTAAGATCTGCTAGCTCGAGATCCTCATCACTAAATAA
pCAGGS-Flag-MDA5-ΔC2	F: GAATTCGAGCTCATCGATGGTACCATGTCGAATGGGTATTCC R: TTAATTAAGATCTGCTAGCTCGAGCTAATCCTCATCACTAAATAAACA
pCAGGS-Flag-MDA5-ΔATP	F: GAATTCGAGCTCATCGATGGTACCATGTCGAATGGGTATTCC R: TTAATTAAGATCTGCTAGCTCGAGCTAATCCTCATCACTAAATAAACA
pCAGGS-Flag-MDA5-ΔCTD	F: GAATTCGAGCTCATCGATGGTACCATGTCGAATGGGTATTCC R: TTAATTAAGATCTGCTAGCTCGAGCTTTTCATTTTCATATTC
pCAGGS-Flag-MDA5-CARD	F: GAATTCGAGCTCATCGATGGTACCATGTCGAATGGGTATTCC R: TTAATTAAGATCTGCTAGCTCGAGGGCAACTTCCATTTGGTA
pCAGGS-Flag-MAVS-ΔCARD	F: GAATTCGAGCTCATCGATGGTACCGTCTACCAGAGCTACCAG R: TTAATTAAGATCTGCTAGCTCGAGGTGCAGACGCCGCCGGTA
pCAGGS-Flag-MAVS-ΔN	F: GAATTCGAGCTCATCGATGGTACCCCAGATGGTGGCCCCCTG R: TTAATTAAGATCTGCTAGCTCGAGGTGCAGACGCCGCCGGTA
pCAGGS-Flag-MAVS-ΔTM	F: GAATTCGAGCTCATCGATGGTACCATGCCGTTTGCTGAAGAC R: TTAATTAAGATCTGCTAGCTCGAGAGGTGAGGGCCTGTGGCA
pCAGGS-Flag-MAVS-N	F: GAATTCGAGCTCATCGATGGTACCATGCCGTTTGCTGAAGAC R: TTAATTAAGATCTGCTAGCTCGAGTGGATTCCTTGGGATGGC
pCAGGS-HA-MDA5	F: GAGCTCATCGATGGTACCATGTCGAATGGGTATTCCACAGACGAGAATTT R: AGATCTGCTAGCTCGAGATCCTCATCACTAAATAAACAGCATTCTGAATA
pCAGGS-HA-MAVS	F: TGCGAATTCGAGCTCATCGATGGTACCATGCCGTTTGCTGAAGACAAGA R: TAATTAAGATCTGCTAGCTCGAGCTAGTGCAGACGCCGCCGGTACA

### Viral infection

For qRT-PCR or immunoblot analysis, cells (2 × 10^5^) were plated 24 h before infection with various viruses at the specified time points. For viral replication assays, peritoneal macrophages were infected with EMCV and VSV for 0, 4, 8, and 12 h. Viral replication was analyzed by qRT-PCR. For mouse infection, six- to eight-week-old age- and sex-matched *Anxa2^+/+^* and *Anxa2^-/-^* littermates were intraperitoneally injected with EMCV (2 × 10^4^ PFU per mouse). For survival experiments, the animals' survival was monitored daily after EMCV infection. Sera from EMCV-infected mice were collected for ELISA analysis at 48 h and 72 h post-infection, and the heart and brain tissues were collected for qRT-PCR, EMCV titers, or histological analysis.

### Luciferase reporter gene assay

Luciferase activities were measured with the Dual-Luciferase Reporter Assay System (Promega), according to the manufacturer’s instructions. Data were normalized for transfection efficiency by dividing the value of Firefly luciferase activity by the value of Renilla luciferase activity.

### RNA extraction and qPCR

Total RNA was extracted using TRIzol reagent (Invitrogen), and the reverse transcription products were amplified using the Agilent-Stratagene MxReal-Time qPCR system with a PrimeScript RT Reagent Kit (Takara). The reverse transcription products were amplified using an Agilent-Stratagene MxReal-Time qPCR system with TB Green Premix Ex Taq II (Tli RNaseH Plus) (Takara) according to the manufacturer’s instructions. Data were normalized to the level of β-actin expression in each individual sample. The qPCR primers are listed in Table 2.

**TABLE 2:** Primers used for qPCR and different virus strains in this study

Gene name	Primers (5′−3′)
Human *Ifn-β1*	F: ATGACCAACAAGTGTCTCCTCC R: GCTCATGGAAAGAGCTGTAGTG
Human *β-actin*	F: CCTTCCTGGGCATGGAGTCCTG R: GGAGCAATGATCTTGATCTTC
Human *Isg56*	F: TCATCAGGTCAAGGATAGTC R: CCACACTGTATTTGGTGTCTAG
Human *Mx1*	F: CTCCGACACGAGTTCCACAA R: GGCTCTTCCAGTGCCTTGAT
Mouse *Ifn-β1*	F: CCCTATGGAGATGACGGAGA R: CTGTCTGCTGGTGGAGTTCA
Mouse *Gapdh*	F: AAATGGTGAAGGTCGGTGTGAAC R: CAACAATCTCCACTTTGCCACTG
Mouse *Isg56*	F: TGCGATCCACAGTGAACAAC R: ACTTCCGGGAAATCGATGAG
Mouse *Mx1*	F: CCTGGAGGAGCAGAGTGACAC R: GGTTAATCGGAGAATTTGGCAA
EMCV	F: TGAGCTTAGACCGATAGA R: GATGCAAACTTTCCCAAC P: AGGTTCAAGCCGCCAAGACA
VSV	F: CAAGTCAAAATGCCCAAGAGTCACA R: TTTCCTTGCATTGTTCTACAGATGG

### IFN sensitivity assay

The cellular supernatants were collected and used to assess their ability to inhibit VSV-GFP replication. Briefly, HEK293T cells, HEK293T-*Anxa2^-/-^* cells, or HEK293T-*Anxa2^-/-^* cells overexpressing ANXA2 were infected with SeV at an MOI of 10. The cell supernatants were collected and UV-deactivated using a 254 nm UV light source for 15 min. The UV-deactivated cell supernatants were then diluted 1:10 in RPMI-1640 medium and added to MDBK cells. After a 24 h pre-treatment, MDBK cells were infected with VSV-expressing GFP at an MOI of 5.0 for 5–8 h. VSV infection was confirmed by observing fluorescence under a UV light source.

### Co-immunoprecipitation and Western blot analysis

Co-immunoprecipitation and Western blot analysis were performed as previously described (34). Briefly, for Co-IP, whole cell extracts were lysed in a lysis buffer (50 mM Tris-HCl, pH 7.4, 150 mM NaCl, 5 mM MgCl_2_, 1 mM EDTA, 1% Triton X-100, and 10% glycerol) containing 1 mM PMSF and 1× protease inhibitor cocktail (Roche). Then, cell lysates were incubated with anti-Flag (M2) beads or Protein G Plus-Agarose immunoprecipitation reagent (Santa Cruz Biotechnology) along with 1 μg of the corresponding antibodies at 4°C overnight on a roller. The precipitated beads were washed five times with cell lysis buffer. For Western blot analysis, equal amounts of cell lysates and immunoprecipitants were resolved by 10%–12% sodium dodecyl sulfate polyacrylamide gel electrophoresis and then transferred to a polyvinylidene difluoride membrane (Millipore). After incubation with primary and secondary antibodies, the membranes were visualized by ECL chemiluminescence (Thermo Fisher Scientific) or an Odyssey two-color infrared fluorescence imaging system (LI-COR).

### Confocal microscopy analysis

HEK293T cells were transfected with the indicated plasmids and then fixed for 20 min in 4% paraformaldehyde in 1× phosphate-buffered saline (PBS) at pH 7.4. The fixed cells were permeabilized for 20 min with 0.3% Triton X-100 in 1× PBS and then blocked in 1× PBS with 10% bovine serum albumin for 30 min. The cells were incubated with the appropriate primary antibodies and then stained with Alexa Fluor 594-labeled goat anti-mouse immunoglobulin G and Alexa Fluor 488-labeled goat anti-rabbit IgG. The subcellular localizations of the indicated proteins were visualized using a Zeiss LSM-880 laser scanning fluorescence microscope (Carl Zeiss AG, Oberkochen, Germany) with a 63× oil-immersion objective lens.

### Generation of *Anxa2*-deficient mice

CRISPR/Cas9 genomic editing was utilized for gene deletion in cell lines and mice, following previously described methods (35). To generate mammalian *Anxa2^-/-^* cells, a single CRISPR guide RNA (sgRNA) sequence targeting the ANXA2 locus in the genome was selected based on specificity scores (http://crispr.mit.edu/). The sgRNA sequence used is as follows: ANXA2 sgRNA, 5′-GCACTGAAGTCAGCCTTATCTGG-3′. This sgRNA sequence was cloned into the pSpCas9 (BB)−2A-GFP plasmid (pX458, Addgene). The construct was then transfected into HEK293T cells individually. Cells expressing GFP were sorted using flow cytometry, and single cells were seeded into 96-well plates. After clonal expansion, ANXA2 protein in different clones was examined through immunoblot analysis. Genomic DNAs from those clones with undetectable ANXA2 protein expression were isolated and subjected to PCR amplification for ANXA2 gene sequencing.

### Histopathology analysis

The lungs of WT and *Anxa2^-/-^* mice infected with VSV were fixed in a 10% formalin neutral buffer solution overnight. The tissues were embedded in paraffin blocks, and then sectioned at a 4-μm thickness and stained with hematoxylin and eosin in accordance with standard procedures. The results were analyzed using light microscopy. Representative views of the lung sections are presented.

### Statistical analysis

Statistical analysis was conducted using an unpaired Student’s *t*-test, a two-tailed Student’s *t*-test, and one-way or two-way ANOVA followed by the Bonferroni post-test. *P*-values less than 0.05 were considered statistically significant. For mouse survival studies, Kaplan-Meier survival curves were generated and analyzed for statistical significance with GraphPad Prism 6.0. Sample sizes were chosen by standard methods to ensure adequate power, and no exclusions, randomization by weight or sex, or blinding was used for animal studies.

## Data Availability

All relevant data are within the article. The data that support the findings of this study are available from the corresponding author upon reasonable request.
